# Comparison of infection and human immune responses of two SARS-CoV-2 strains in a humanized hACE2 NIKO mouse model

**DOI:** 10.1038/s41598-023-39628-y

**Published:** 2023-08-01

**Authors:** Kylie Su Mei Yong, Danielle E. Anderson, Adrian Kang Eng Zheng, Min Liu, Sue Yee Tan, Wilson Wei Sheng Tan, Qingfeng Chen, Lin-Fa Wang

**Affiliations:** 1 Institute of Molecular and Cell Biology (IMCB), Agency for Science, Technology and Research (A*STAR), 61 Biopolis Drive, Proteos, Singapore, 138673 Singapore; 2 Programme in Emerging Infectious Diseases, Duke-NUS Medical School, Singapore, Singapore; 3 Department of Microbiology and Immunology, University of Melbourne at the Peter Doherty Institute for Infection and Immunity, Melbourne, Australia; 4 Department of Microbiology and Immunology, Yong Loo Lin School of Medicine, National University of Singapore, Singapore, Singapore; 5 Singhealth Duke-NUS Global Health Institute, Singapore, Singapore

**Keywords:** Immunology, Microbiology

## Abstract

The COVID-19 pandemic has sickened millions, cost lives and has devastated the global economy. Various animal models for experimental infection with SARS-CoV-2 have played a key role in many aspects of COVID-19 research. Here, we describe a humanized hACE2 (adenovirus expressing hACE2) NOD-SCID IL2Rγ^−/−^ (NIKO) mouse model and compare infection with ancestral and mutant (SARS-CoV-2-∆382) strains of SARS-CoV-2. Immune cell infiltration, inflammation, lung damage and pro-inflammatory cytokines and chemokines was observed in humanized hACE2 NIKO mice. Humanized hACE2 NIKO mice infected with the ancestral and mutant SARS-CoV-2 strain had lung inflammation and production of pro-inflammatory cytokines and chemokines. This model can aid in examining the pathological basis of SARS-CoV-2 infection in a human immune environment and evaluation of therapeutic interventions.

An outbreak of Severe Acute Respiratory Syndrome coronavirus 2 (SARS-CoV-2) was first identified in December 2019 in Wuhan, China^1^. Coronavirus disease 2019 (COVID-19) was declared a pandemic on March 11, 2020 and to date, there have been over 765 million cases and 6.9 million deaths attributed to the virus^2^.

As SARS-CoV-2 is constantly mutating, quick adaptation and effective treatments are needed^3,4^. To understand disease pathogenesis and assess therapeutics, animal models are required^5–8^. Angiotensin-converting enzyme-2 (ACE2) is used by SARS-CoV-2 as a cellular receptor to enter target cells^9^. SARS-CoV-2 is able to effectively enter human cells via human ACE2 (hACE2), but is unable to infect mice via mouse ACE2 (mACE2)^10–12^. In addition, human immune cells are essential for modulating responses to SARS-CoV-2 and the lack of an observable response in mice is a major hurdle in the analysis of disease pathogenesis and therapeutic invention^13–15^.

Multiple methods to create COVID-19 mouse models have been utilized, including modification of SARS-CoV-2 spike protein^5^, sequentially passaging SARS-CoV-2 in mouse lung tissue^16–18^, creating transgenic mice where hACE2 is under the control of a tissue-specific promoter such as K18 or cytomegalovirus (CMV)^19–23^, substituting mACE2 with hACE2^24,25^ and transduction of adenovirus (AdV) or adeno-associated virus expressing hACE2 into the respiratory tract of mice^20,26^. These models can support viral replication, but the human immune system remains absent.

It is observed that there is a myriad of disease manifestation among SARS-CoV-2 infected patients^27^. The use of humanized mice with variations in immune responses can represent diverse cohorts of people. Therefore, to generate a mouse model with a human immune system that supports SARS-CoV-2 replication, we created an AdV expressing hACE2 and packaged the construct into plasmids using helper RNAs encoding capsid and envelope proteins to produce hACE2. Adenovirus infects both replicating and non-replicating cells, can be utilized to deliver large transgenes, encode for proteins without integrating into the host genome and is an efficient vector for gene delivery^28^.

A humanized mouse model susceptible to SARS-CoV-2 infection was created through exogenous delivery of hACE2 with AdV. Humanized hACE2 (adenovirus expressing hACE2) NIKO mice were infected with either an ancestral SARS-CoV-2 (referred to as WT herein) or the variant SARS-CoV-2-∆382 (referred to as Δ382 herein) to analyze the effect of an ORF8 deletion in mice with a human immune system^4,29^.

## Results

### Generation and characterization of humanized hACE2 NIKO mice

**Figure 1:**
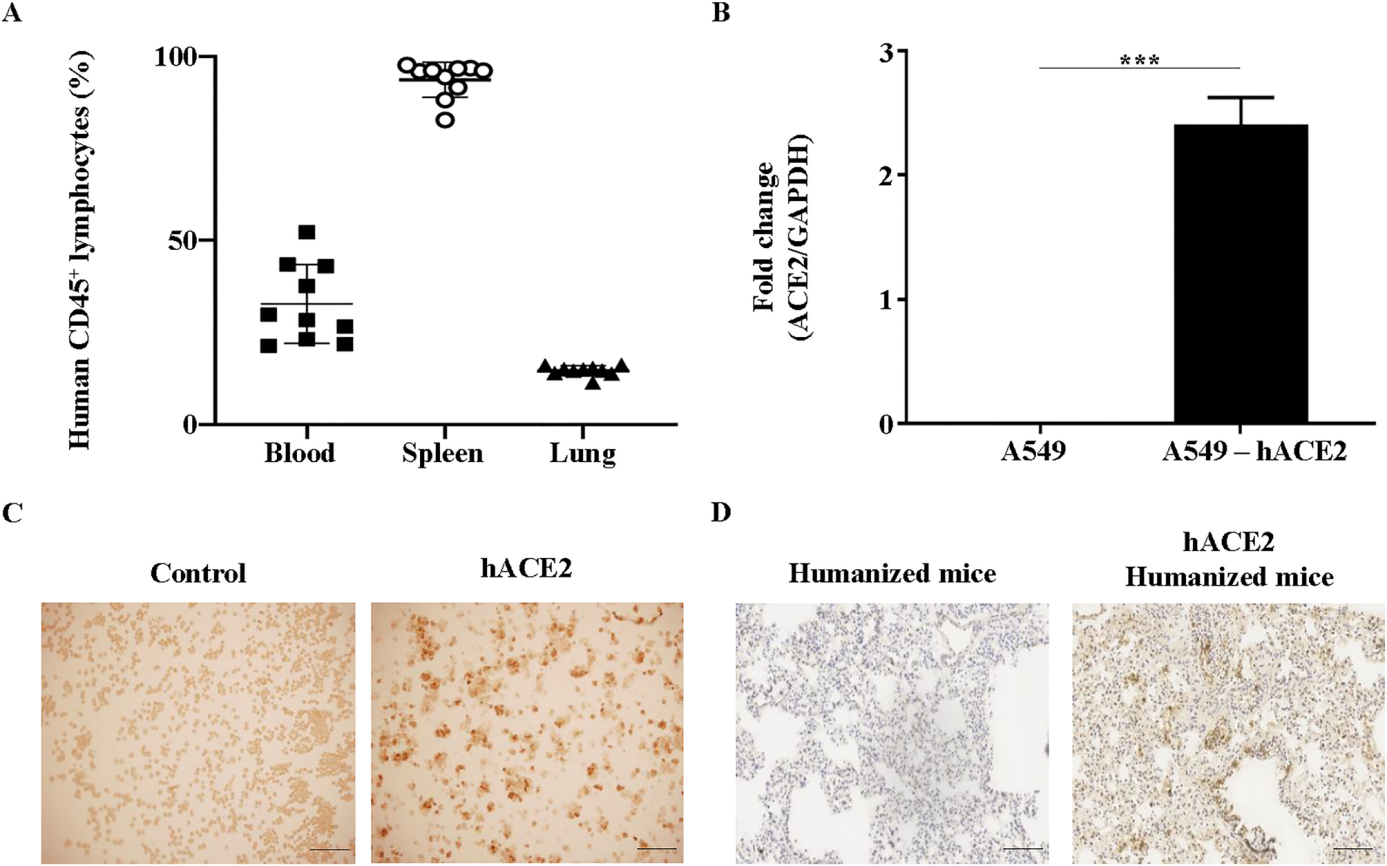
Generation of humanized hACE2 NIKO mice. (**A**) Percentages of human CD45^+^ lymphocytes in blood, spleen and lungs of mice at 12-weeks post-transplantation of CD34^+^ hematopoietic stem cells. Data representative of 2 independent in vivo experiments. Data are represented as mean ± SEM. (**B**) and (**C**) Validation of human ACE2 (hACE2) expression in human lung cancer cell line A549 by RT-PCR. Data representative of 2 independent in vitro experiments. (**B**) Validation of human ACE2 (hACE2) expression in human lung cancer cell line A549 by immunohistochemistry. Scale bar represents 100 µm and images are representative from two independent in vitro experiments (**C**). (**D**) Confirmation of hACE2 expression in humanized hACE2 NIKO mouse lungs by immunohistochemistry. Scale bar represents 200 µm and images are representative from 2 independent in vivo experiments.

Humanized mice were created by engrafting CD34^+^ stem cells into humanized hACE2 NIKO mice. Twelve-weeks post-transplantation, blood samples were collected and analyzed for human reconstitution by flow cytometry (FACS). Additional control mice were sacrificed, and human immune cell reconstitution was evaluated. Human immune populations were present in the blood, spleen, and lung of humanized mice at variable levels (Fig. 1A).

Helper RNA encoding capsid and envelope proteins were used to package AdV expressing hACE2 into plasmids to generate hACE2 AdV. After intratracheally inoculating mice with hACE2 AdV, the presence of hACE2 expression was confirmed in lung cells transduced with hACE2 AdV by qPCR (Fig. 1B) and immunohistochemistry (Fig. 1C). Immunohistochemistry confirmed hACE2 expression in mouse lung tissue (Fig. 1D).

**Figure 2:**
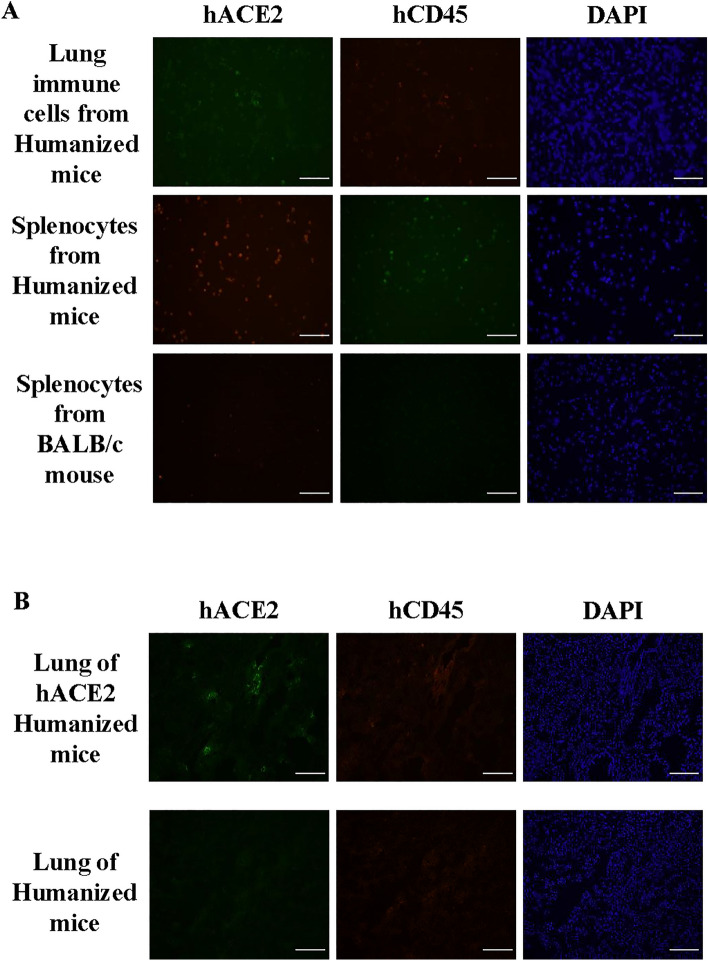
Expression of hACE2 in immune cells in humanized mice. (**A**) Lung and spleen immune cells from humanized hACE2 NIKO and BALB/c mouse were stained with hACE2, hCD45 and DAPI. Scale bar represents 100 µm and images are representative from 2 independent in vivo experiments. (**B**) Lung tissue from humanized hACE2 NIKO and untreated control humanized mice were stained with hACE2, hCD45 and DAPI. Scale bar represents 100 µm and images are representative from 2 independent in vivo experiments.

As humanized mice were reconstituted with human immune cells, we further characterized if immune cells express ACE2 receptor. Lung and spleen immune cells from humanized and BALB/c mice were enriched and directly stained with hACE2, hCD45 and DAPI. Our data confirmed that the some of CD45^+^ human immune cells express ACE2 receptors (Fig. 2A). To understand the in vivo distribution of hACE2 in hACE2 AdV transduced humanized mice, lung sections from hACE2 humanized mice and control humanized mice were stained with hACE2, hCD45 and DAPI. It was shown that hACE2 was expressed mainly in the lung stromal cells of humanized mouse transduced with hACE2 AdV (Fig. 2B), while the contribution of hACE2^+^hCD45^+^ cells is minimal.

### Humanized hACE NIKO mice support SARS-CoV-2 infection

**Figure 3:**
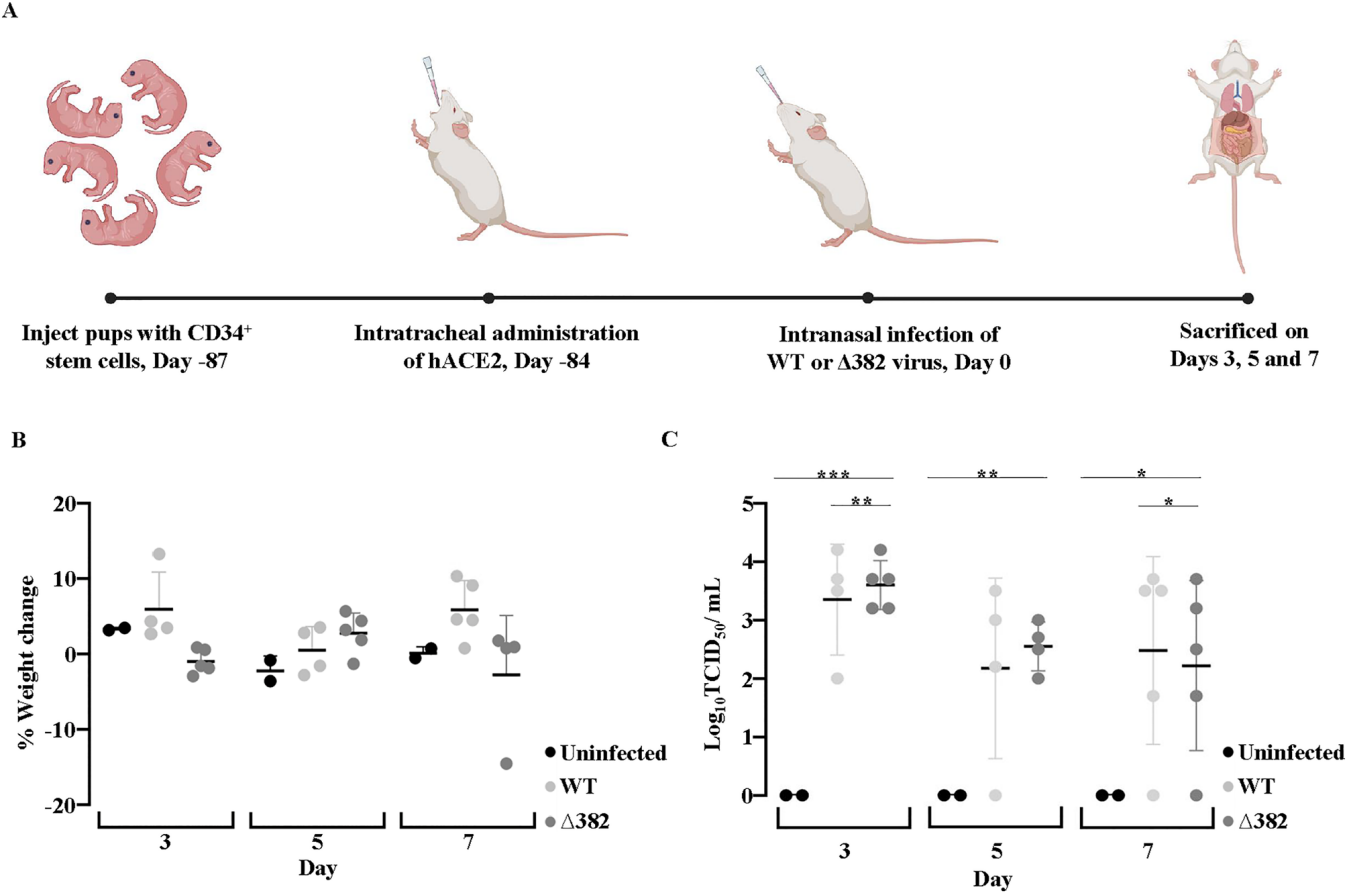
Humanized mice transduced with hACE2 AdV support SARS-CoV-2 infection. (**A**) Schematic illustration showing generation and infection of humanized hACE2 NIKO mouse model with WT or ∆382 viruses. Measurement of body weight (**B**) and virus titre (**C**) in uninfected (UI) controls, WT and ∆382 infected humanized hACE2 NIKO mice. Data representative of 2 independent in vivo experiments. Data are represented as mean ± SEM. Two-tailed Mann–Whitney U test; (*p < 0.05, **p < 0.01, ***p < 0.001).

**Figure 4:**
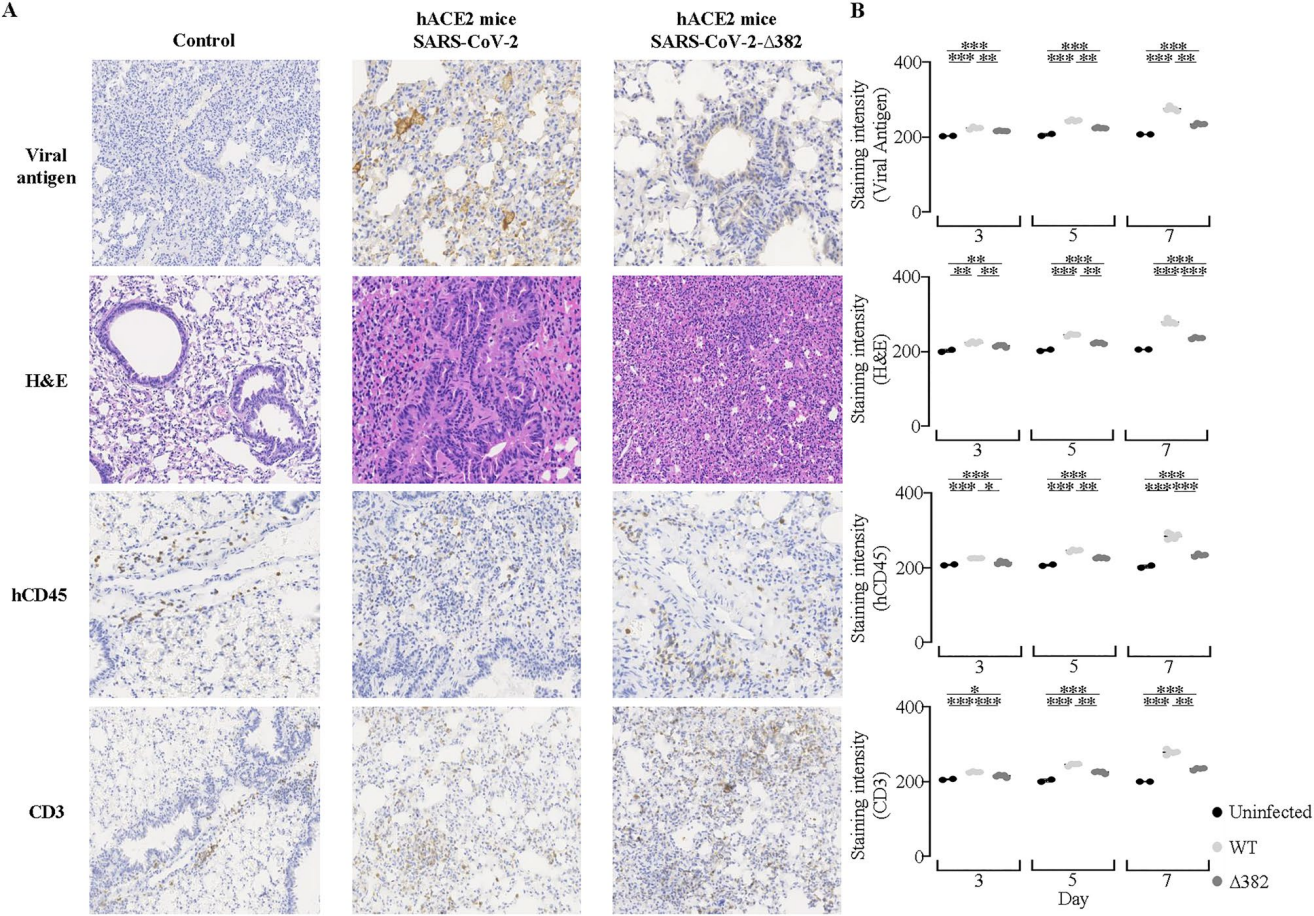
Humanized mice transduced with hACE2 AdV exhibits inflammatory responses when infected with SARS-CoV-2. Peak responses of (**A**) Viral antigen (spike protein), human immune cell infiltration, human CD45^+^ lymphocytes and CD3^+^ T cells are present in humanized hACE2 NIKO mice infected with SARS-CoV-2 or SARS-CoV-2-∆382 at day 7. Scale bar represents 100 µm and images are representative from 2 independent in vivo experiments. (**B**) Staining intensity in humanized hACE2 NIKO mice infected with ∆382 as compared to control and WT virus as observed in histological stains of Viral antigen (spike protein), human cell infiltration, human CD45^+^ lymphocytes and CD3^+^ T cells.

To create optimal humanized mice, 84 days is needed for human immune cells to expand and reconstitute immunodeficient mice. At 84 days old, mice were intratracheally administered with hACE2 AdV and used for experiments 3 days after inoculation. Therefore, NIKO pups were injected with CD34^+^ stem cells 87 days prior (Day -87) to the start of the experiment. Day 0 indicates the start of the experiment where humanized hACE2 NIKO mice were infected with either WT or Δ382 virus. At 84 days before (Day -84) the start of the experiment, humanized hACE2 NIKO mice were intratracheally administered with hACE2 AdV. At the start of the experiment at Day 0, humanized hACE2 NIKO mice were infected with either WT or Δ382 virus and groups of mice were sacrificed at 3, 5 and 7 dpi (Fig. 3A). There was no significant weight loss for the duration of infection and no difference between groups (Fig. 3B). Viral replication in the lungs peaked at 3 dpi in mice infected with WT and Δ382 (Fig. 3C). Virus infection in the lung was confirmed by histology. Viral antigen was present in lung sections in humanized mice transduced with hACE2 AdV (Fig. 4A).

### Immune cells infiltrated the lungs of infected humanized mice

To characterize human immune responses to SARS-CoV-2 infection in humanized mice, histology from day 3, 5 and 7 were stained. Day 7 where peak responses were observed was used to identify cell types by staining for hCD45 and CD3^+^ T cells (Fig. 4A). Analysis of lungs sections from humanized hACE2 NIKO mice sacrificed at day 7 and stained with H&E revealed infiltration of immune cells and lung damage. Lung sections of humanized hACE2 NIKO mice infected with WT or Δ382 virus had hCD45^+^ cells in the lungs. The presence of hCD45^+^ leukocytes suggest that infection was mediated by human immune cells. We further demonstrated the infection of both strains induced human CD3^+^ T cell infiltration in the lung. In addition, staining intensity in humanized hACE2 NIKO mice infected with WT and ∆382 virus as observed in histological stains of viral antigen (spike protein), human cell infiltration, human CD45^+^ lymphocytes and CD3^+^ T cells showed that humanized hACE2 NIKO mice can support WT and Δ382 infection (Fig. 4B).

### Infected humanized mice exhibit inflammatory responses

**Figure 5:**
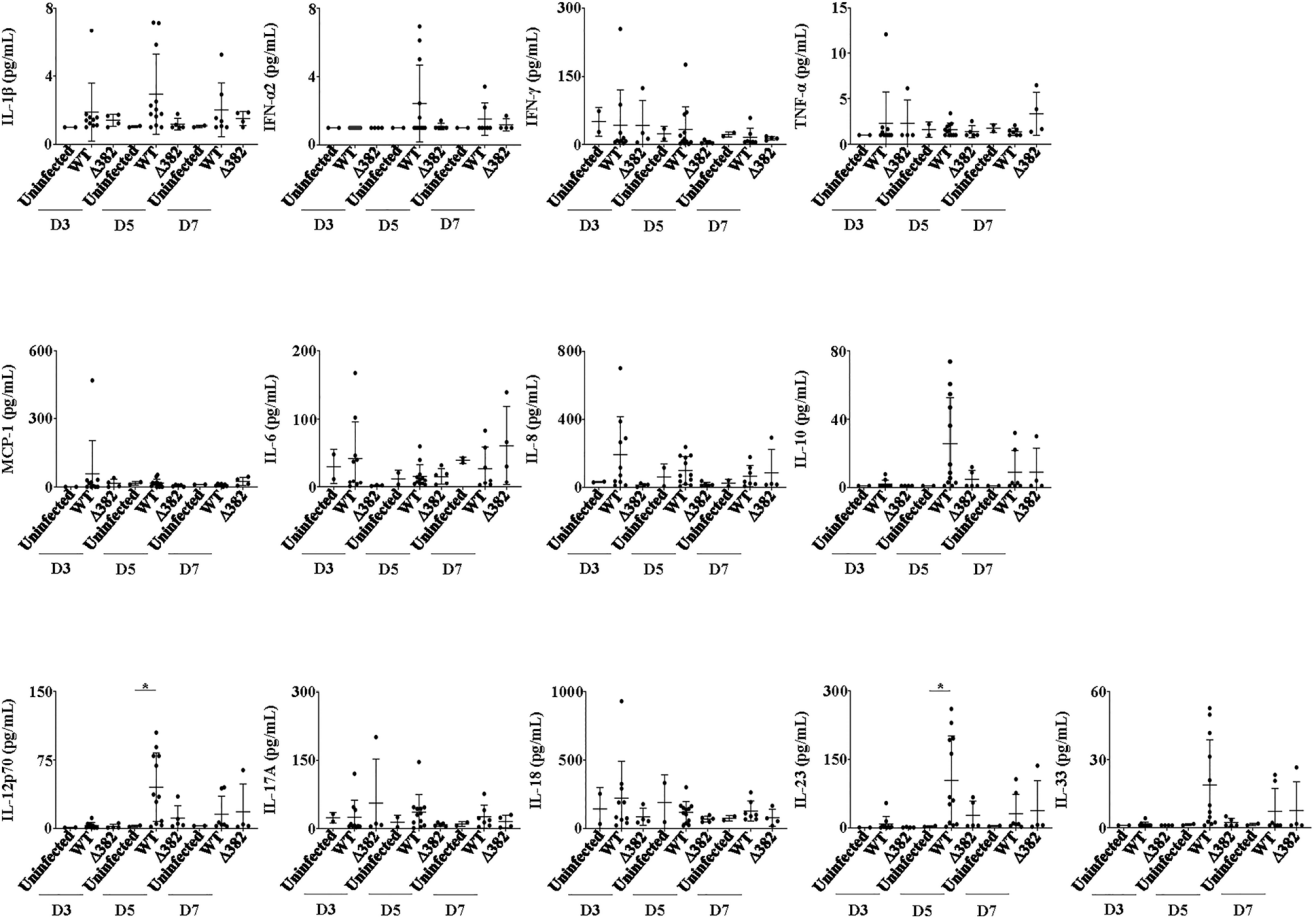
Analysis of cytokine and chemokine responses in infected humanized mice. Human-specific cytokine and chemokine release of IL-1β, IFN-α, IFN-γ, TNF-α, MCP-1 (CCL2), IL-6, IL-8 (CXCL8), IL-10, IL-12p70, IL-17A, IL-18, IL-23, and IL-33 were measured in the plasma of humanized mice administered with WT, ∆382 and UI controls at day 3 (D3), day 5 (D5) and day 7 (D7). Data representative of 2 independent in vivo experiments. Data are represented as mean ± SEM. Two-tailed Mann–Whitney U test; (*p < 0.05, **p < 0.01, ***p < 0.001).

Cytokine storm and release is known to be responsible for severe syndrome in patients. The presence of human immune system in humanized mice is essential for production of human cytokines and chemokines in WT or ∆382 infection. To determine if humanized hACE2 NIKO mice were able to recapitulate clinical observations, sera from mice were analyzed using a LEGENDplex™ panel. Human pro-inflammatory cytokines, immunosuppressive cytokine and chemokines such as IL-1β (WT and ∆382 mice on 3, 5 and 7 dpi), IFN-α2 (WT mice on 5 and 7 dpi), IFN-γ (WT mice on 5 dpi), TNF-α (∆382 mice on 7 dpi), IL-6 (WT mice on 3, 5 and 7 dpi), IL-8 (CXCL8) (WT mice on 3, 5 and 7 dpi), IL-10 (WT and ∆382 mice on 5 and 7 dpi), IL-10 (WT and ∆382 mice on 5 and 7 dpi), IL-12p70 (WT and ∆382 mice on 5 and 7 dpi), IL-17A (WT mice on 3, 5 and 7 dpi, ∆382 mice on 3 dpi), IL-18 (WT mice on 3 and 7 dpi), IL-23 (WT and ∆382 mice on 5 and 7 dpi) and IL-33 (WT mice on 5 dpi, ∆382 mice on 7 dpi) were produced in varying amounts and timepoints, albeit in lower concentrations as compared to reported patient values (Fig. 5)^4,30,31^.

## Discussion

To overcome infectious disease outbreaks, accurate testing platforms that can be deployed quickly are needed to understand disease pathogenesis and screen therapeutics rapidly. Specificity of non-human animal models used to evaluate disease pathogenesis and test therapeutics are variable and may underestimate therapeutic interaction with human immune cells^32,33^. To design and test therapeutics safely, a simple and accessible animal model with human immune cells is needed.

Humanized mouse models produced in our laboratory have previously been utilized to study a myriad of infectious diseases, such as dengue fever and hepatitis. Humanized mouse infected with Dengue virus (DENV) developed viremia, rash, fever, thrombocytopenia, released anti-DENV IgM, IgG and produced cytokines similar to patients^34^. When infected with hepatitis B or C virus, humanized mouse developed inflammation of the liver, leukocyte infiltration, fibrosis, cirrhosis with increased levels of cytokines, similar to human infections^35,36^. By responding to infection, generating inflammation, and developing pathological changes, these models greatly assisted the understanding of human immune responses to infectious diseases.

In this work, we established a humanized hACE2 NIKO mouse model which contains both human immune cells and the hACE2 receptor to potentially evaluate disease pathogenesis, human immune responses, and outcomes to therapeutics. We prove that although human immune cells naturally express certain levels of hACE2, they are not sufficient to support large extent of viral replications in vivo in humanized mice. Hence, an introduction of hACE2 AdV to humanized mouse models is essential and greatly improves virus infection and replication. Even though there was no significant weight loss during infection, viral replication peaked at 3 dpi in mice infected with WT or Δ382. In addition, viral antigen can be detected in lung sections of humanized mice transduced with hACE2 AdV.

Most importantly, this novel model allows us to examine human immune responses and pathological changes to various strains of SARS-CoV-2 infection. Humanized hACE2 NIKO mice infected with SARS-CoV-2 developed pathological damage in the lungs which includes inflammatory cell infiltration and tissue injury, while humanized hACE2 NIKO mice without human immune system reconstitution only supported infection with no inflammation and pathological changes. Corresponding to inflammation and immune cell infiltration in lung, human cytokines and chemokines such as IL-1β, IFN-α2, IFN-γ, TNF-α, IL-6, IL-8 (CXCL8), IL-10, IL-12p70, IL-17A, IL-18, IL-23 and IL-33 were found in systemic circulation in infected mice in varying amounts and timepoints, although in lower concentrations as compared to reported patient values.

These results confirm the importance of human immune responses in SARS-CoV-2 pathogenesis, which opens a window for the study of mechanisms and evaluation of therapeutics against inflammation during infection and post-infection. Human immune cells may also express ACE2 receptors, making them a target for SARS-CoV-2, which may result in cell activation and production of more human pro-inflammatory cytokines^14,37–40^.

We noticed that although human immune responses were detected in humanized mice, the extent of pathological changes and levels of human cytokines produced were not as robust as observed in patient cohorts. Therefore, the current setting of humanized mice requires optimization. For example, myeloid cells are the main source of pro-inflammatory cytokines, such as IL-6, as observed in COVID-19 patients. However, levels of myeloid cells are relatively low in humanized mice (∼5–8% in blood) compared to human (∼40–70%), hence the amount of cytokine and chemokine production may be disproportioned to levels observed in patients.

It has been shown that the development and function of specific human immune cell types such as human natural killer (NK) cells and myeloid cells are less optimal in this humanized mouse system^41,42^. To further improve SARS-CoV-2 responses, particularly the levels of pro-inflammatory cytokines and lung pathological changes, the humanized hACE2 NIKO mouse model can be optimized by boosting mice with a cocktail of human-specific growth factors/cytokines such as IL-3, IL-7, IL-15, M-CSF and GM-CSF, which have been successfully used in other disease models^43–46^. These growth factors will not only support critical immune subsets but also activate human-specific responses toward pathogens.

Further, considering the deficiency of IL2Rγ in mouse immune cells, it has been shown that the residual mouse immune system has impaired function^47^. However, we still cannot exclude the participation of mouse immune cells during COVID-19 infection in this model. This will be studied in our improved model. To add on, with improved humanized mice, we may be able to study a range of factors including accessory proteins. Understanding the functions of these accessory proteins may provide key insights into the mechanisms underlying SARS-CoV-2 infection and aid in the development of effective therapeutics and vaccines^48,49^.

In summary, our current study demonstrates the utility of humanized mice in conjunction of virus receptor expression. By manipulating the receptor humanized mice expresses for specific pathogens, humanized mice can be a powerful tool for rapid establishment of animal models to study human immune responses and pathogenesis of new pathogens. For example, humanized mice can be engineered to express dipeptidyl-peptidase 4 (DPP4) for studying MERS-CoV^50^.

## Conclusion

In conclusion, immune cells infiltration, inflammation, lung damage and pro-inflammatory cytokines and chemokines was observed in humanized hACE2 NIKO mice infected with WT and ∆382. This model can aid in examining the pathological basis of SARS-CoV-2 infection in a human immune environment and evaluation of therapeutic interventions.

Our study demonstrates the utility of humanized mice in conjunction of virus receptor expression. By manipulating the receptor humanized mice expresses for specific pathogens, humanized mice can be a powerful tool for rapid establishment of animal models to study human immune responses and pathogenesis of new pathogens. For example, humanized mice can be engineered to express dipeptidyl-peptidase 4 (DPP4) for studying MERS-CoV. A simple and accessible animal model with human immune cells is needed to design and test therapeutics safely. By responding to infection, generating inflammation, and developing pathological changes, these models can greatly assist the understanding of human immune responses to infectious diseases.

## Methods

### Human cord blood samples

Human cord blood (CB) samples were obtained from healthy males and females aged 16–23 weeks through STEMCELL Technologies with informed and written consent. This study was approved by the Institutional Review Board (IRB) at Agency for Science, Technology and Research (A*STAR). Experimental procedures were conducted in accordance with IRB approval (CIRB Ref: 2012/064/B). All methods were performed in accordance with the relevant guidelines and regulations. The purity of the CD34^+^ cells was 90–99% as determined by flow cytometry. Two cord blood samples were used for this study. Experimental mice were chosen randomly, regardless of sex and reconstitution level.

### Mice

NOD-SCID IL2Rγ^−/−^ (NIKO) mice were generated and bred in the animal facility at A*STAR, Biological Resource Centre (BRC). Neonate mice were sub-lethally irradiated (100 rads) within 72 h of birth and infused with 1 × 10^5^ human CD34^+^ hematopoietic stem cells (HSCs) via intra-hepatic injection. At 12-weeks post-transplantation, flow cytometry was used to determine human immune cell reconstitution levels in the peripheral blood of mice. BALB/c mice were purchased from InVivos. Male and female mice were used in this study. Mice were bred and housed under specific pathogen free conditions. All animal experimental procedures were conducted in accordance with the guidelines of Agri-Food and Veterinary Authority and the National Advisory Committee for Laboratory Animal Research of Singapore and were approved by the International Animal Care and Use Committee (IACUC), A*STAR (BRC #151060).

### Control and hACE2 mice

Recombinant replication-deficient E1, E3-deleted type 5 Adeno-X™ Adenoviral System was used (Takara, USA). The cDNA encoding human ACE2 or EGFP was used to prepare recombinant adenovirus expressing hACE2 or EGFP. Adenovirus expressing hACE2 or EGFP was propagated in 293A cells and purified by Caesium chloride (CsCl) discontinuous density gradient centrifugation. Titre of the vectors was in the range of 1.1–2.0 × 10^11^ pfu/ml as determined by Adeno-X Rapid Titre Kit (Takara, USA).

### Cells and viruses

Two SARS-CoV-2 isolates were used in this study: (1) SARS-CoV-2, an ancestral Wuhan strain (hCoV-19/Singapore/2/2020, GISAID accession ID: EPI_ISL_407987); and (2) SARS-CoV-2-∆382, an ancestral Wuhan strain containing a 382-nt deletion (hCoV-19/Singapore/12/2020, GISAID accession ID: EPI_ISL_414378). Both viruses were isolated from COVID-19 patients in Singapore, as reported previously^4^ and referred to as wild-type (WT) and mutant (∆382), respectively, herein. Virus was propagated using Vero-E6 cells cultured with DMEM supplemented with 5% fetal bovine serum (FBS) and 100 units/ml penicillin and 100 µg/ml streptomycin (P/S) at 37 °C, 5% CO_2_. Cell culture supernatants were harvested, centrifuged and aliquoted once cytopathic effect (CPE) was observed. Vero-E6 cells were infected with virus stocks to determine the tissue culture infective dose (TCID_50_/ml). Vero-E6 cells in 96-well plates were used for titrations. Virus stocks were serially diluted in DMEM supplemented with 5% FBS and P/S. Cells were infected with 100 µL of sample dilution in quadruplicate. Cells were incubated at 37 °C and 5% CO_2_ for 4 days. Following incubation, wells with CPE were recorded and the cell-free virus titre (TCID_50_/mL) for each sample were determined by limited dilution.

### Infection of mice with WT and ∆382 SARS-CoV-2

Three to five mice were assigned to each group. Mice were infected with 5 × 10^4^ TCID intranasally following sedation with isoflurane. Body weight was monitored on days 3, 5 and 7. Mice were euthanized by CO_2_ overdose and lungs were taken for titration and stored at − 80 °C until homogenized in 1 ml DMEM-5 and titrated by limiting dilution as described above. Blood was collected from the submandibular vein bleed on the last day of the study. Blood was collected into a tube containing heparin and centrifuged at 3000 rpm for 15 min to obtain plasma.

The experiments were approved by the SingHealth Institutional Animal Care and Use Committee of the SingHealth Experimental Medicine Centre (SEMC). Infection studies were performed under BSL3 containment in the Duke-NUS Medical School ABSL3 facility.

### Cytospin, histology and immunohistochemistry

For cytospin, mononuclear cells (MNCs) were isolated from the lung and spleen by standard procedures. The spleen was pressed through a 70 μm mesh and debris removed by centrifugation at 1500 rpm for 5 min. Supernatant containing MNCs were collected, washed once in PBS, and resuspended in 40% Percoll (MilliporeSigma, USA) diluted in RPMI medium 1640. The cell suspension was carefully overlaid onto 70% Percoll and centrifuged at 2500 rpm for 20 min. Mononuclear cells were collected from the interphase and washed twice in PBS. Lung was minced and added to a medium containing 0.05% collagenase (MilliporeSigma, USA), 0.01% DNase I (MilliporeSigma, USA), and incubated at 37 °C for 20 min. The lung sample was filtered through a 70 μm mesh and MNCs were isolated by Percoll centrifugation as described above. Mononuclear cells from lung and spleen were counted and 10^4^ cells resuspended in 100 μl of 2% FCS—PBS were added to each cytospin cassette and centrifuged at 1000 rpm for 5 min. Cells were cytospun onto Poly-L-Lysine-coated slides, air-dried and fixed with 4% paraformaldehyde (PFA).

Humanized mouse lungs were collected and fixed in 4% PFA prior to removal from the ABSL3 facility. Lungs were then embedded in paraffin and processed into sections. PFA-fixed paraffin sections (5 μm thickness) were dewaxed by melting for 30 min at 65 °C, rinsed in xylene twice for 5 min, and rehydrated in water–ethanol solutions containing decreasing percentages of ethanol. Lungs from humanized mice not infected with WT or Δ382 virus was collected from euthanized mice and embedded in Tissue-Tek® O.C.T™ compound (Sakura Finetek, USA) and snap-frozen. Cryosections of 5 μm were obtained from frozen liver tissue. To determine tissue morphology, sections were stained with Haematoxylin–eosin (Gill 2 Haematoxylin and Eosin Y alcoholic; Thermo Sandon, UK) following standard procedures. For Immunohistochemistry, mouse lungs were subjected to heat-mediated antigen retrieval with sodium citrate (pH6 or pH9), incubated with anti-human CD3 (pH9; 1:50) (Abcam, UK), CD45 (pH6; 1:50) (Abcam, UK) and SARS-CoV-2 anti-spike antibody (pH6; 1:400) (GeneTex, USA) antigen stained with SuperPicture 3rd Gen IHC Detection Kit (Life Technologies, USA) according to manufacturer’s instructions. Cytospun splenocytes and lung cells were stained with anti-ACE2 (1:100) (Abcam, UK), human CD45 (1:40) (Abcam, UK), Chicken anti-Mouse IgG (H + L) Cross-Adsorbed Secondary Antibody, Alexa Fluor 488 (1:200) (Thermo Fisher Scientific, USA), Goat anti-Rabbit IgG (H + L) Cross-Adsorbed Secondary Antibody, Alexa Fluor 546 (1:200) (Thermo Fisher Scientific, USA) and cover-slipped with Prolong Gold anti-fade reagent containing DAPI (Invitrogen). Histopathological images were acquired using Axio Scan. Z1 slide scanner (Zeiss, Germany) and analyzed using Zen 2 (blue edition; Zeiss) software. Windows 10 and ImageJ (Version 1.53) was used to quantitate histological images. Briefly, images were converted into 8-bit, backgrounds subtracted, and biologically relevant areas were measured.

### Cytokine and chemokine protein quantification

As LEGENDplex™ provides higher sensitivities and a broader dynamic range than traditional ELISA methods, a premixed LEGENDplex™ Human Inflammation Panel (13-plex) (Biolegend, USA) was used to measure plasma cytokine and chemokine levels. The 13 cytokines and chemokines assayed simultaneously include IL-1β, IFN-α, IFN-γ, TNF-α, MCP-1 (CCL2), IL-6, IL-8 (CXCL8), IL-10, IL-12p70, IL-17A, IL-18, IL-23, and IL-33. Immunoassay procedures were performed according to manufacturer’s instructions. Data was acquired using a LSR II flow cytometer (BD Biosciences, USA) with FACSDiva software, and analysis was performed using LEGENDplex™ Data Analysis software (Biolegend, USA) based on standard curves plotted through a five-parameter logistic curve setting.

### Statistical analysis

Statistical analysis was performed using GraphPad Prism 5.0 software (GraphPad Software Inc., USA). The correlation strength between the variables was assessed using the Spearman's rank correlation coefficient. Pairwise comparison was performed using two-tailed Mann Whitney U test/ two-way Analysis of variance (ANOVA), p value less than 0.05 is considered statistically significant.

### ARRIVE guidelines

This study is reported in accordance with ARRIVE guidelines.

## Data Availability

All data generated or analyzed during this study are included in this published article (and Supplementary Information files).
